# Beyond Trisomy 21: Phenotypic Variability in People with Down Syndrome Explained by Further Chromosome Mis-segregation and Mosaic Aneuploidy

**DOI:** 10.4172/2472-1115.1000109

**Published:** 2016-03-31

**Authors:** Huntington Potter

**Affiliations:** Department of Neurology, and Linda Crnic Institute for Down Syndrome, Rocky Mountain Alzheimer’s Disease Center, University of Colorado Anschutz Medical Center, USA

**Keywords:** Down syndrome, Alzheimer’s disease, Mitosis, Aneuploidy, Aβ peptide, Mosaicism, Phenotypic variation

## Abstract

Phenotypic variability is a fundamental feature of the human population and is particularly evident among people with Down syndrome and/or Alzheimer’s disease. Herein, we review current theories of the potential origins of this phenotypic variability and propose a novel mechanism based on our finding that the Alzheimer’s disease-associated Aβ peptide, encoded on chromosome 21, disrupts the mitotic spindle, induces abnormal chromosome segregation, and produces mosaic populations of aneuploid cells in all tissues of people with Alzheimer’s disease and in mouse and cell models thereof. Thus, individuals exposed to increased levels of the Aβ peptide should accumulate mosaic populations of aneuploid cells, with different chromosomes affected in different tissues and in different individuals. Specifically, people with Down syndrome, who express elevated levels of Aβ peptide throughout their lifetimes, would be predicted to accumulate additional types of aneuploidy, beyond trisomy 21 and including changes in their trisomy 21 status, in mosaic cell populations. Such mosaic aneuploidy would introduce a novel form of genetic variability that could potentially underlie much of the observed phenotypic variability among people with Down syndrome, and possibly also among people with Alzheimer’s disease. This mosaic aneuploidy theory of phenotypic variability in Down syndrome is supported by several observations, makes several testable predictions, and identifies a potential approach to reducing the frequency of some of the most debilitating features of Down syndrome, including Alzheimer’s disease.

## Introduction

Almost all people with Down syndrome carry a complete or nearly complete extra copy of chromosome 21 [1,2]. The resulting alterations in expression, both from genes on chromosome 21 itself and from genes on other chromosomes affected, for example, by transcription factors encoded on chromosome 21, lead to a wide range of phenotypic differences between people with Down syndrome and those of the typical population [1,3,4]. Curiously, there is also a high degree of phenotypic variability among people with Down syndrome. For example, only a very small percentage of people with Down syndrome develop childhood leukemia, whereas a much larger percentage of people with Down syndrome, but not all, are born with heart defects requiring surgery [5–8]. Thus, understanding the underlying causes of the phenotypic variability among people with Down syndrome is of equal scientific interest and medical importance as understanding the phenotypic variability between people with Down syndrome and the typical population. Indeed, if it were possible to understand the mechanism underlying the variability among people with Down syndrome, it might be possible to develop interventions directed at increasing the likelihood of more beneficial characteristics and reducing the likelihood or severity of challenging ones.

It is widely accepted among Down syndrome researchers that changes in gene expression caused by trisomy 21 are influenced by other inherited alleles and by the environment, thus leading to phenotypic variability that is similar to that observed in the typical population. However, recent work from our laboratory has identified another likely contributor to phenotypic variability in people with Down syndrome that may be of equal or greater consequence. Specifically, we have found that the A peptide, encoded by the amyloid precursor protein (APP) gene that resides on chromosome 21, can induce large, permanent changes in the genome with potentially wide-ranging effects on gene expression, and, thus, on phenotypes in people with Down syndrome.

The A peptide is produced via proteolytic cleavage of the amyloid precursor protein by β-secretase and β-secretase (presenilin) and is the key component of the Alzheimer’s disease pathogenic pathway. According to the ‘Amyloid Cascade Hypothesis,’ increased levels of the Aβ peptide lead to phosphorylation of the microtubule stabilizing protein Tau, which aggregates into neurofibrillary tangles that cause neuronal damage and neurodegeneration [9–13]. Some years ago, we proposed and then demonstrated that people with Alzheimer’s disease develop mosaic trisomy 21 and other aneuploidy as a consequence of mitotic defects leading to chromosome mis-segregation. The trisomy 21 cells, in turn, would contribute to Alzheimer’s disease pathology and dementia via the same mechanism that trisomy 21 leads to Alzheimer’s disease neuropathology by the age of 40 years and a greatly increased risk of Alzheimer’s disease dementia in people with Down syndrome and complete trisomy 21, but at a later age due to the modulating effect of the majority euploid cells in typical Alzheimer’s disease [14–16]. This result has been replicated and extended in several laboratories, and, indeed, the accumulation and specific degeneration of the almost 30% of neurons that are aneuploid in the brain can account for 90% of the neuronal cell loss observed at autopsy in typical Alzheimer’s disease ([17–24]; for reviews see [25,26]).

In our investigations of the mechanisms by which aneuploidy arises in Alzheimer’s disease, we discovered that Aβ-42, either added exogenously or produced endogenously due to APP overexpression or due to expression of familial Alzheimer’s disease-causing mutant forms of the APP or presenilin genes, directly induces mitotic spindle abnormalities, chromosome mis-segregation, and aneuploidy both in mouse and cell culture models of Alzheimer’s disease [27,28]. In subsequent studies, we discovered that Aβ-42 binds to and inhibits several microtubule-dependent motor proteins that are essential for normal mitotic spindle structure and function [29].

The finding that the Aβ peptide induces chromosome mis-segregation and aneuploidy leads us to hypothesize that the excess Aβ peptide produced in people with Down syndrome throughout their lifetimes will lead to stochastic chromosome mis-segregation and the accumulation of mosaic aneuploidy in tissues that include dividing cells and/or cells that can re-enter the cell cycle in response to stress or to cell loss/damage. Furthermore, the Aβ peptide-induced chromosome mis-segregation-a stochastic process-will lead to different levels of aneuploidy for the various chromosomes in each individual and in each tissue within an individual. This highly genetically variable population of cells will exhibit different patterns of gene expression in individual tissues and different individuals, resulting in the observed phenotypic variability.

This mosaic aneuploidy theory of phenotypic variability in Down syndrome makes testable predictions that our own work and searches of the literature indicate have already been partly satisfied.

## Prediction 1

There should be evidence of chromosome mis-segregation/aneuploidy in people with Down syndrome that affects multiple chromosomes, including potentially chromosome 21 itself.

## Prediction 2

Because Alzheimer’s disease is at least in part a cell cycle disease in which the generation and specific loss of aneuploid neurons in the brain accounts for 90% of the neuronal cell loss at autopsy, a person with trisomy 21 Down syndrome but with only two copies of the APP gene should not exhibit mosaic aneuploidy and may not develop Alzheimer’s disease.

We will consider each of these predictions in view of current knowledge.

1) First, all available data are consistent with the hypothesis that people with Down syndrome are prone to undergoing additional chromosome mis-segregation beyond trisomy 21 and including changes in their trisomy 21 status. For example, Percy et al. [30] and Jenkins et al. [31] found that many people with DS progressively lose their third copy of chromosome 21 in up to 4% of their peripheral blood lymphocytes, and that they also exhibit increased levels of mosaic aneuploidy for other chromosomes. Furthermore, several researchers found an increased frequency of micronuclei (a measure of chromosome mis-segregation, chromosome breakage/damage, and genomic instability) in buccal cells [32–36] and in peripheral blood lymphocytes [37] in both younger and aged people with DS.

Such divergent levels of aneuploidy and genomic instability may underlie the observed phenotypic variation in people with Down syndrome, particularly with regard to the variation in their age of onset of Alzheimer’s disease dementia. Specifically, although every adult with Down syndrome will have the characteristic neuropathology of Alzheimer’s disease by the age of 30–40 years, the average age of onset of Alzheimer’s disease dementia varies greatly, with ~25% starting to show signs of dementia in their early thirties, and with 25–50% having a more delayed onset, or not developing dementia at all even by age >60 years (for reviews, see [3,38]).

2) Multiple investigations have revealed a cell cycle defect in Alzheimer’s disease [26], including a defect in mitosis that leads to chromosome mis-segregation, trisomy 21, and other forms of aneuploidy, as we predicted [15]. For example, flourescence *in situ* hybridization (FISH) was used to measure aneuploidy, and specifically trisomy 21, in primary fibroblasts from Alzheimer’s disease patients and age-matched control individuals [14,16]. Our analysis of thousands of fibroblasts from 27 AD and 13 control individuals showed that the fibroblasts from the Alzheimer’s disease patients were more than twice as likely (p = 0.007) to exhibit trisomy 21 relative to the fibroblasts from the age-matched control individuals. Notably, the increased frequency of trisomy 21 cells in fibroblasts from Alzheimer’s disease patients was independent of age.

We also found that chromosome mis-segregation was associated with all types of Alzheimer’s disease, including sporadic and familial Alzheimer’s disease (i.e., patients carrying a mutation in either of the presenilin (PS1 or PS2) genes or in the APP gene. Finally, we also detected increased levels of chromosome 18 aneuploidy in fibroblasts from Alzheimer’s disease patients, indicating that the cell cycle defect leading to chromosome mis-segregation was not specific to chromosome 21, although it appeared to be associated with chromosome 21 most often, possibly due to trisomy 21 cells being less prone to dying *in vivo* compared to other types of aneuploid cells.

Chromosome mis-segregation in patients with sporadic Alzheimer’s disease, particularly involving, but not restricted to, chromosome 21, has been confirmed in blood lymphocytes [20,21], in buccal cells [23], and in brain neurons [17–19,22,24] As discussed, our further investigations showed that mutations in the APP or presenilin genes that cause familial Alzheimer’s disease, and exposure to increased levels of the Aβ peptide, the product of the cleavage of the APP protein by the presenilin-containing β secretase and the β-secretase (BACE), induce chromosome mis-segregation and aneuploidy in mouse models and cell culture models of AD [27–29].

The significance of the close to 30% trisomy 21 and other aneuploidy in the Alzheimer’s disease brain and the specific loss of aneuploid neurons in Alzheimer’s disease is reinforced by the fact that all aneuploid calls are prone to apoptosis [39–43], which we have also found to be true in the brain.

Similar to amyloid deposits, the increased levels of aneuploidy in the Alzheimer’s disease brain appear to be likely due to the overproduction of the Aβ peptide and its oligomers. Overproduction of Aβ may arise, for example, as a consequence of familial AD-causing mutations in the APP or presenilin genes or by an inflammation-induced increase in APP translation [44,45], whereas an increased conversion of the Aβ peptide to toxic oligomers is promoted by the catalytic activity of apolipoprotein-E, especially the apoE4 variant [46]. These mechanisms would most certainly be exacerbated by the third copy of the APP gene in any trisomy 21 cells that arise. Therefore, if the third copy of the APP gene were eliminated in trisomy 21 cells of people with Down syndrome, then Aβ-induced aneuploidy, apoptosis, and possibly Alzheimer’s disease symptoms should not arise. Indeed the few reports of people with Down syndrome who carry only two copies of the APP gene due to a deletion of one copy of the APP gene on one of their three copies of chromosome 21 indicate a lack of Alzheimer-like dementia even at age 60+ [47]. On the other hand, if the tau pathology in people with trisomy 21 Down syndrome partly arises by a mechanism that is dependent on the extra copy of chromosome 21, but independent of Aβ, then some neuronal loss and cognitive decline may still be evident, which future studies may clarify. We would further predict that because people with Down syndrome with only two copies of the APP gene lack the aneugenic activity associated with excess Aβ peptide, they will not accumulate additional mosaic aneuploidy with age and will therefore show less variability (i.e., they will occupy the middle of the distribution curve) for other Down syndrome-related clinical phenotypes, including cognition, some of which may be more or less pronounced in the vast majority of people with Down syndrome who have three copies of the APP gene and thus an increased propensity for mosaic aneuploidy beyond trisomy 21.

The mosaic aneuploidy hypothesis of phenotypic variability in Down syndrome makes additional predictions that we are in the process of testing. For example, the brains of people with Down syndrome who die in their 50s and 60s without evidence of Alzheimer’s disease dementia may be somehow resistant to the aneugenic (and, therefore, cell death-inducing) activity of Aβ and may, thus, show decreased levels of neuronal aneuploidy and apoptosis compared to individuals who die with both Down syndrome and Alzheimer’s disease dementia. People with Down syndrome who appear resistant to Alzheimer’s disease dementia may also exhibit less variability in their other clinical phenotypes. Interestingly, the results of our preliminary studies show that typical aged individuals without Down syndrome who died with extensive Alzheimer’s disease brain pathology, yet had no signs of Alzheimer’s disease dementia, show little evidence of trisomy 21 or other aneuploidy in their brain neurons.

## Conclusion

In sum, the ability of the Alzheimer’s disease-associated Aβ peptide to induce mitotic spindle abnormalities, chromosome mis-segregation, and mosaic aneuploidy can lead to variable levels of aneuploidy in people with trisomy 21 Down syndrome, and, therefore, to variations in gene copy number and expression. Such variations in gene expression between different, genetically mosaic tissues and individuals may underlie the wide phenotypic variability that is observed among different people with Down syndrome.
